# Optimizing production of Fc-amidated peptides by Chinese hamster ovary cells

**DOI:** 10.1186/s12896-015-0210-4

**Published:** 2015-10-16

**Authors:** Kristina Carlson, Steven C. Pomerantz, Omid Vafa, Michael Naso, William Strohl, Richard E. Mains, Betty A. Eipper

**Affiliations:** Department of Neuroscience, University of Connecticut Health Center, 263 Farmington Avenue, Farmington, CT 06030-3401 USA; Biologics Research, Biotechnology Center of Excellence, Janssen Research & Development, LLC, Spring House, PA 19477 USA; Department of Molecular Biology and Biophysics, University of Connecticut Health Center, Farmington, CT 06030 USA

**Keywords:** CHO cell, Glucagon-like peptide 1, Peptide YY, Neuromedin U, Mass spectrometry

## Abstract

**Background:**

Amidation of the carboxyl terminal of many peptides is essential for full biological potency, often increasing receptor binding and stability. The single enzyme responsible for this reaction is peptidylglycine α-amidating monooxygenase (PAM: EC 1.14.17.3), a copper- and ascorbate-dependent Type I membrane protein.

**Methods:**

To make large amounts of high molecular weight amidated product, Chinese hamster ovary (CHO) cells were engineered to express exogenous PAM. To vary access of the enzyme to its substrate, exogenous PAM was targeted to the endoplasmic reticulum, *trans-*Golgi network, endosomes and lysosomes or to the lumen of the secretory pathway.

**Results:**

PAM was equally active when targeted to each intracellular location and assayed in homogenates. Immunocytochemical analyses of CHO cells and a pituitary cell line demonstrated that targeting of exogenous PAM was partially successful. PAM substrates generated by expressing peptidylglycine substrates (glucagon-like peptide 1-Gly, peptide YY-Gly and neuromedin U-Gly) fused to the C-terminus of immunoglobulin Fc in CHO cell lines producing targeted PAM. The extent of amidation of the Fc-peptides was determined by mass spectrometry and amidation-specific enzyme immunoassays. Amidation was inhibited by copper chelation, but was not enhanced by the addition of additional copper or ascorbate.

**Conclusions:**

Peptide amidation was increased over endogenous levels by exogenous PAM, and targeting PAM to the endoplasmic reticulum or *trans-*Golgi network increased peptide amidation compared to endogenous CHO PAM.

## Background

Amidated peptides act as hormones, neuromodulators and autocrine growth factors [1, 2]. Each amidated peptide is synthesized from a peptidylglycine precursor through the actions of peptidylglycine α-amidating monooxygenase (PAM: EC 1.14.17.3). In mice engineered to lack the *Pam* gene, peptide amidating activity is not detectable and embryos die at mid-gestation [3]. PAM contains two catalytic domains, peptidylglycine α-hydroxylating monooxygenase (PHM) and peptidyl-α-hydroxyglycine α-amidating lyase (PAL). The enzymes act sequentially, first converting the peptidylglycine substrate into a short-lived peptidyl-α-hydroxyglycine intermediate and then cleaving the C-N bond to produce the amidated peptide and glyoxylate [2].

The active site of PHM contains two copper residues, each of which is essential for activity. Copper is not tightly bound to PHM and no other metal can substitute for it. ATP7A, a P-type ATPase, transports the copper it receives from cytosolic copper-binding chaperones into the lumen of the secretory pathway, where the copper is available to PHM [2, 4, 5]. Mutations in human *ATP7A* cause Menkes Disease, a lethal disorder characterized by copper deficiency [6, 7]. Mice bearing a mutation in the *Atp7a* gene display similar symptoms and survive for less than two weeks after birth. Among the many deficits observed in these mice is the inability to produce normal levels of amidated peptides [8]. Chelation of copper *in vitro* or *in vivo* also leads to a reduced ability to produce amidated peptides [9].

The reaction catalyzed by PHM is still not fully understood, but requires two single electron transfer steps. Ascorbic acid (vitamin C) is present at high levels in the secretory pathway and is generally the source of the reducing equivalents needed to support peptide amidation [2]. In the absence of ascorbic acid in cell culture systems, peptide amidation fails to occur and other single electron donors or reducing agents (e.g. NADH, NADPH, dithiothreitol, dopamine), cannot fully substitute for ascorbate [10].

Previous studies of the production of amidated peptides in cell lines have had mixed results. Using transfected CHO and COS7 cells, Takahashi et al. [11] found very efficient amidation of salmon calcitonin (C-terminal Pro-NH_2_), while Hayashi et al. [12] reported that amidation of gastrin (C-terminal Phe-NH_2_) was efficient in CHO cells but not in COS7 cells. These results are puzzling, since peptides terminating with –Phe-Gly are far better substrates for PAM that peptides terminating with –Pro-Gly, using test tube assays and purified enzyme [1]. Johansen et al. [13] showed that amidation of NPY (C-terminal Tyr-NH_2_), another excellent PAM substrate, only proceeded to 50–80 % completion in different CHO cell lines. Work using neuroendocrine lines which express prohormone convertases along with PAM consistently always shows complete amidation after transfection of preprohormone precursor cDNAs [14–18]. Thus, it is difficult to predict which peptide precursors will be efficiently amidated in which cell lines, especially if the goal is to achieve essentially 100 % amidation without extraneous or unwanted endoproteolytic cleavages.

In an attempt to prolong the half-lives of amidated peptides, we engineered CHO cells to produce Fc-peptidylglycine fusion proteins in the absence and presence of exogenous PAM; both soluble and integral membrane forms of PAM were tested for their ability to support Fc-peptidylglycine fusion protein amidation in CHO cells [19]. The extent of amidation observed varied from 25 to 90 % for different Fc- peptidylglycine substrates, but the expression of exogenous PAM always increased the amidation of Fc- peptidylglycine substrates [19]. The extent of amidation never reached 100 %, which would be essential for many pharmacotherapeutic applications. It is clear that PAM activity is rate-limiting for peptide amidation in CHO cells, since increasing PAM increased amidation [19], while decreasing PAM lowered the extent of amidation [20].

Since PHM requires both copper and ascorbate to function, we explored three ways to improve the ability of CHO cells to secrete amidated Fc-fusion peptides. First, we used known targeting signals to try to localize integral membrane PAM to different subcellular locations in CHO cells expressing an Fc-GLP1-Gly fusion protein. Second, we added exogenous copper or a copper chelator to the culture medium to vary the availability of copper. Third, we added exogenous ascorbate to the culture medium to see if an increase in reducing equivalents would improve Fc-peptidylglycine amidation.

## Results

### Design of targeting vectors

The isoforms of PAM identified in rat, mouse, human and Chinese hamster encode similar soluble and integral membrane proteins. Each isoform includes an N-terminal signal sequence, but the largest isoforms also include a transmembrane domain, generating type I integral membrane proteins. The trafficking of rat PAM1 has been studied extensively in both endocrine cells, which generate soluble PHM from membrane PAM, and in cells which lack the proteases required to make this cleavage. PAM1 cycles between the *trans-*Golgi network (TGN) and the plasma membrane, returning to the TGN after endocytosis [21]. When expressed in endocrine cells, the PHM and PAL catalytic cores are efficiently packaged in secretory granules; when expressed in CHO cells, both active enzymes are secreted into the medium.

**Fig. 1:**
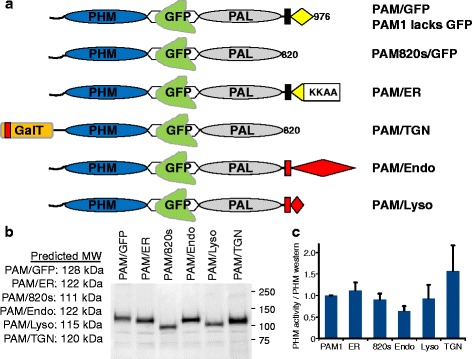
Targeted PAM proteins. **a**. Diagram of PAM1 and GFP-tagged targeted PAM proteins. The regions of ERGIC-53, Galactosyl Transferase 1, cation independent mannose-6-phosphate receptor and LAMP1 used to redirect rat PAM/GFP are indicated. PAM820s/GFP was truncated immediately after the catalytic core of PAL. **b**. pEAK Rapid cells, a derivative of hEK293 cells, were transiently transfected with vectors encoding the indicated proteins. After 24 h, cell extracts were prepared using 20 mM NaTES, 10 mM mannitol, 1 % TX-100, pH 7.4; aliquots were subjected to Western blot analysis and visualized using an antibody to the PHM domain. **c**. PHM activity was measured in each cell lysate and normalized to the amount of PHM protein detected on the Western blot; the PHM activity/PHM protein ratio for PAM/GFP was set to 1.0 for each of three experiments; error bars show the standard deviation. None of the differences were significant

We used well characterized trafficking signals to try to target PAM to different subcellular locations in CHO cells producing an engineered PAM substrate, Fc-AP-GLP1-Gly [19]. To facilitate identification of cells expressing PAM and analysis of trafficking, we used EGFP-tagged rat PAM (Fig. 1a). Since we wanted to use the same tag for each targeting vector, EGFP was inserted into the linker region that separates PHM from PAL in PAM1; 55 amino acids of the linker region were replaced by EGFP, with no deleterious effect on either enzymatic activity [22]. The paired basic cleavage site present in the linker region, which allows endoproteolytic cleavage of PAM1 in neurons and endocrine cells, was eliminated in PAM/GFP. For comparison, a soluble GFP-tagged PAM protein was generated by truncating PAM/GFP after the catalytic core of PAL (PAM820s/GFP).

Well characterized targeting signals known to direct proteins to the endoplasmic reticulum (ER), TGN, endosomes (Endo) and lysosomes (Lyso) were used (Fig. 1a). The cytosolic domain of PAM was replaced by the cytosolic domain of ERGIC-53, generating PAM/ER [23]. GalT, a type II integral membrane protein, is targeted to the TGN by its N-terminal transmembrane and juxtamembrane region [24]; the signal sequence and pro-region of soluble PAM820s/GFP were replaced by this region of GalT, generating PAM/TGN, a putative type II membrane protein. For endosomal targeting, the transmembrane and cytosolic domains of PAM were replaced with the corresponding regions of the cation independent mannose-6-phosphate receptor in PAM/Endo [25]. The transmembrane and cytosolic domains of LAMP1 were used in a similar manner to create PAM/Lyso [26].

To evaluate the catalytic activity of the targeted PAM/GFP proteins, each was expressed transiently. Cell extracts were prepared for analysis of protein integrity (Fig. 1b) and PHM catalytic activity (Fig. 1c). Western blot analysis using an antibody to the PHM domain revealed a single major protein of the expected molecular mass in each lysate. To compare the specific activities of the targeted proteins, PHM activity in each lysate (pmol product/mg/h) was normalized to the amount of PHM protein (arbitrary OD units/mg); data for each targeted PAM protein were normalized to the value for PAM/GFP analyzed at the same time (Fig. 1c). Normalized to PHM protein, the catalytic activities of the targeted PAM proteins did not differ significantly.

### Analysis of cells expressing targeted PAM proteins

**Fig. 2:**
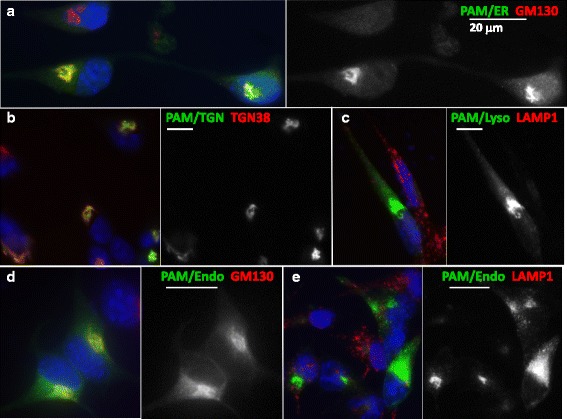
AtT-20 cells expressing targeted PAM constructs. **a**. AtT-20 cells were fixed and permeabilized 24 h after transfection; targeted PAM proteins were visualized using antibody to GFP and a FITC-tagged secondary antibody (green in merged images and white in panels showing GFP alone); subcellular markers were visualized using a Cy3-tagged secondary antibody (red) and nuclei were stained using Hoechst (blue). Cells expressing PAM/ER were stained for GM130 (**a**); three cells are shown, but only two were transfected. Cell expressing PAM/TGN were stained for TGN38 (**b**). Cells expressing PAM/Lyso were stained for LAMP1 (**c**). Cells expressing PAM/Endo were stained for GM130 (**d**) or LAMP1 (**e**). Epifluorescence images were taken using a 60X oil immersion lens; scale bar, 20 μm

The goal of this work was to express Fc-fusion peptides in cells with no prohormone convertases (CHO cells), but there are few good markers of intracellular organelles in CHO cells, while pituitary cells have been studied extensively. Since so much is known about PAM trafficking in pituitary cells [21, 27], each GFP-tagged targeted PAM protein was transiently expressed in AtT-20 corticotrope tumor cells, where its localization could be compared to that of PAM/GFP and established markers for cis-Golgi (GM130), TGN (TGN38), secretory granules (ACTH) and lysosomes (LAMP1). PAM/ER accumulated in the perinuclear region (Fig. 2a). GFP and GM130 staining overlapped extensively, but examination of the merged image revealed that the patterns were not identical (Fig. 2a); PAM/ER staining was also distinguishable from TGN38 staining (Fig. 2b) and the small amount of diffusely distributed PAM/ER staining was distinct from staining for secretory granule granules or lysosomes. Much like PAM/ER, PAM/TGN was localized to the perinuclear region, overlapping extensively with GM130 and TGN38 (Fig. 2b). Examination of the merged images suggested that PAM/TGN staining in the perinuclear region overlapped more with TGN38 than with GM130. PAM/Lyso staining concentrated in the perinuclear area and in vesicles distributed throughout the cytosol; based on their failure to stain with antisera to LAMP1, these vesicles were not lysosomes (Fig. 2c). Based on their localization patterns in AtT-20 cells, the trafficking signals appended to PAM were only partially successful in altering its subcellular localization. Significantly more vesicular staining was observed in cells expressing PAM/Endo (Fig. 2d). Vesicular staining of PAM/Endo was not coincident with staining for LAMP1 (Fig. 2e) or ACTH (not shown). In the perinuclear region, PAM/Endo staining was coincident with TGN38 staining (Fig. 2e), but extended beyond the structures enriched in TGN38. The fact that both luminal domains of PAM are efficiently packaged into secretory granules, with no added routing information [28], presumably limits the extent to which membrane PAM can be re-directed.

### Analysis of stably transfected CHO cell lines expressing the Fc-GLP1-Gly fusion protein and a targeted PAM protein

Since each targeting vector generated an active PAM protein, we next attempted to generate CHO cell lines stably expressing a targeted PAM protein along with substrate by introducing the targeted PAM protein into CHO cells stably expressing Fc-GLP1-Gly. The sequence of GLP1-Gly was appended to the C-terminus of the Fc region of human IgG1, separated by an (Ala-Pro)_10_ linker and a human rhinovirus protease cleavage site (LEVLFQ/GP) [19]. We obtained stable CHO cell lines expressing the Fc-GLP1-Gly fusion protein and three of the targeted PAM proteins, but were unable, despite multiple attempts, to derive a stable line expressing PAM/Lyso. Since PAM/GFP localization resembled that of PAM1, a previously generated CHO line expressing Fc-GLP1-Gly and PAM1 was analyzed for comparison [19].

**Fig. 3:**
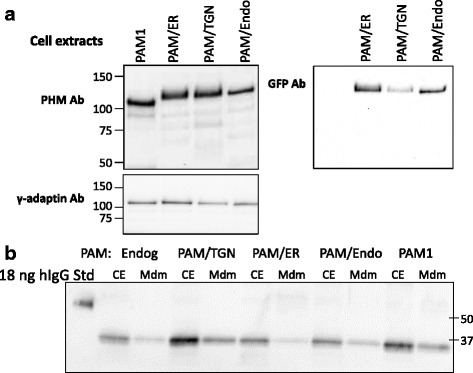
Stably transfected CHO cell lines expressing targeted PAM proteins. **a**. Western blots for PHM, GFP and γ-adaptin. **b**. Western blot for Fc in cell extracts (CE; 15 μg protein) and spent media; human IgG was analyzed as a control

Western blot analysis of cell extracts revealed the presence of proteins of the expected mass; the targeted PAM proteins were recognized by antibodies to both PHM and GFP (Fig. 3a). Cell lysates prepared in buffer containing 1 % TX-100 were assayed for PHM and PAL activity (Table 1). Enzymatic activity was five to 10-fold higher in the targeted PAM lines and in the previously established PAM1 line than in the Fc-GLP1-Gly fusion protein cell line, which expresses endogenous CHO PAM. PHM secretion rates for the different lines were determined by assaying PHM activity in spent medium and ranged from 5 %/h for PAM/ER and PAM/TGN to 11 %/h for PAM/Endo and 22 %/h for PAM1. Cell content and secretion of Fc-GLP1-Gly fusion protein were evaluated by Western blot (Fig. 3b) and quantified (Table 1); Fc content and secretion rate were similar in each of the lines.

**Table 1:** PAM and AP-GLP1-Fc: expression levels and secretion rates. The indicated cell lines were extracted as described in the legend to Fig. 1 and assayed for PHM and PAL activity; data from multiple dilutions of at least 3 independent extracts were averaged and standard errors reported. PHM secretion ( % content/h) was calculated by assaying PHM activity in a 16 h medium collection. Fc levels in cell extracts and spent media were determined by Western blot and used to calculate cell content of Fc and Fc secretion rate

Cell line	PHM pmol/μg/h	PAL pmol/μg/h	PHM secretion % content/h	Fc, μg/mg cell protein	Fc secretion % content/h
AP-GLP1 (Parental line)	0.17 ± 0.01	0.8 ± 0.3	18 ± 4.8	1.3 ± 0.04	8.0 ± 1.4
AP- GLP1	1.8 ± 0.15	17.1 ± 2.0	4.6 ± 1.1	1.2 ± 0.11	6.9 ± 2.4
PAM/ER					
AP-GLP1	1.2 ± 0.2	10.5 ± 3.4	4.5 ± 1.2	2.3 ± 0.5	17.8 ± 1.7
PAM/TGN					
AP-GLP1	0.90 ± 0.12	17.7 ± 4.8	11.0 ± 2.5	1.4 ± 0.1	10.0 ± 2.6
PAM/Endo					
AP-GLP1	1.5 ± 0.21	13 ± 10	22 ± 2.8	2.3 ± 0.2	20.4 ± 2.3
PAM-1					

### Localization of targeted PAM proteins in stable CHO cell lines

**Fig. 4:**
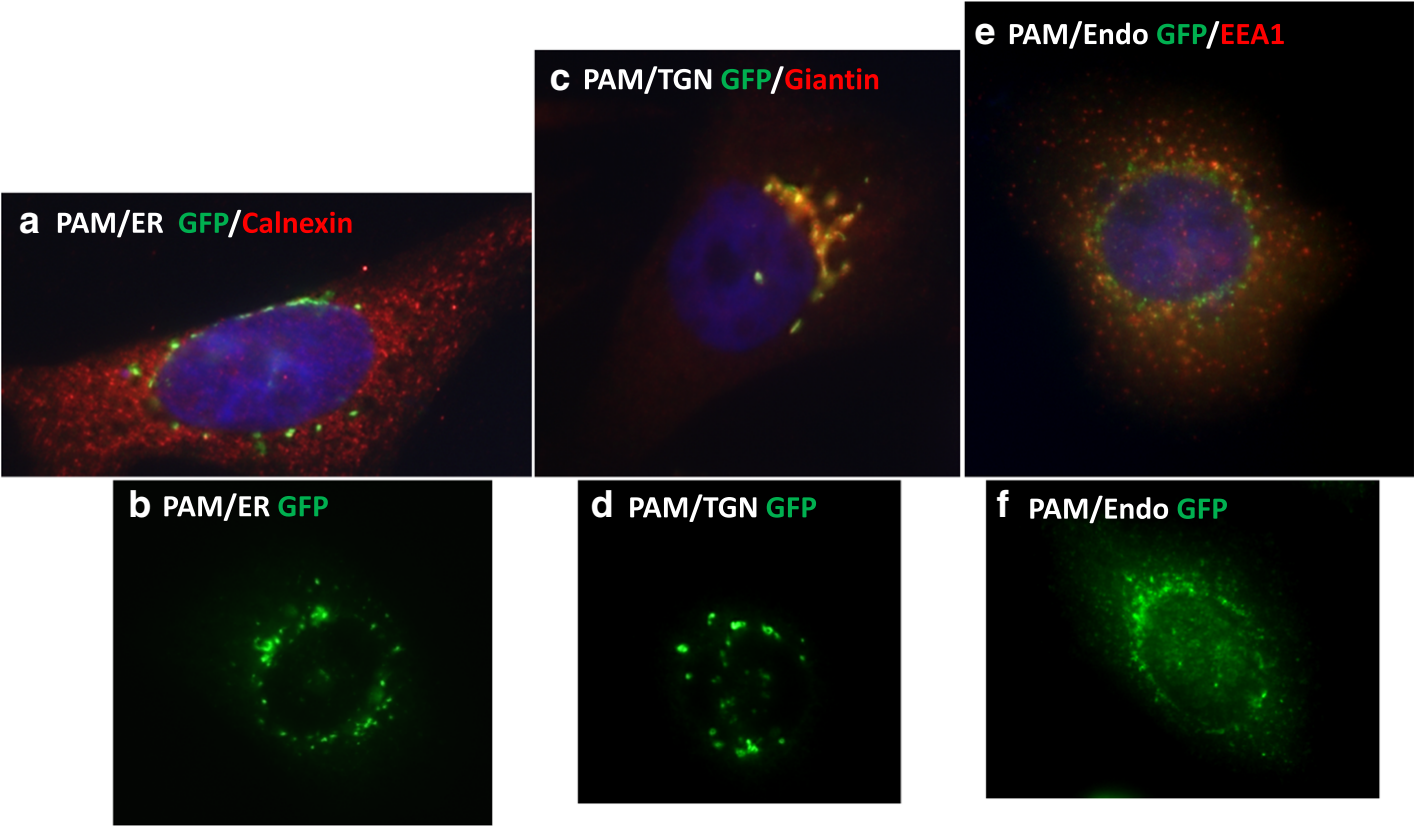
Subcellular localization of targeted PAM proteins. The indicated CHO cell lines were fixed, permeabilized and stained simultaneously with antisera to GFP (visualized using a FITC-tagged antibody) and to the indicated subcellular markers (visualized using a Cy3-tagged antibody). Cells expressing PAM/ER were stained for GFP and calnexin (**a**) or GFP alone (**b**). Cells expressing PAM/TGN were stained for GFP and giantin (**c**) or for GFP alone (**d**). Cells expressing PAM/Endo were stained for GFP and EEA1 (**e**) or for GFP alone (**f**). Epifluorescence images were taken using a 60X oil immersion lens

The steady state localization in CHO cells of each targeted PAM protein was compared to that of recognized subcellular markers for the endoplasmic reticulum (calnexin), cis-Golgi (giantin) and endosomes (early endosomal antigen 1, EEA1). PAM/ER accumulated in puncta surrounding the nucleus; staining for calnexin revealed the expected widely distributed reticular pattern, with little overlap with PAM/ER (Fig. 4a, b). Staining for PAM/ER showed significant overlap with compartments containing giantin, but little overlap with EEA1 containing compartments (not shown). PAM/TGN also accumulated in puncta surrounding the nucleus, overlapping compartments containing giantin (Fig. 4c, d); very little dispersed vesicular staining was observed. Although PAM/Endo also accumulated in puncta surrounding the nucleus (Fig. 4e), its distribution was more widespread, with tiny puncta of staining distributed throughout the cell. Co-localization of PAM/Endo and EEA was readily apparent in these widely dispersed vesicular structures (Fig. 4f); little overlap was observed with calnexin positive compartments, and only partial overlap with giantin positive compartments was observed (not shown). Unlike the separation observed between PAM/TGN or PAM/Endo and a cis-Golgi marker (GM130) in the transiently transfected AtT-20 cells, extensive overlap of both PAM/TGN and PAM/Endo with a cis-Golgi marker (giantin) was observed in the stably transfected CHO cell lines.

### Analysis of Fc-GLP1 fusion protein glycosylation and amidation

Fc-GLP1 fusion proteins secreted by the PAM1 and targeted PAM CHO cell lines were purified using HiTrap Protein A cartridges. The Fc region of the Fc-peptidylglycine substrate includes a single N-linked oligosaccharide. Evaluation of the glycan distribution of Fc-AP-GLP1 purified from CHO cells expressing PAM/TGN, PHMcc (PAM monooxygenase catalytic core; [2] ), PAM1 and PAM/Endo was determined by reverse-phase HPLC-MS intact mass analysis (not shown). The patterns were complex, perhaps indicating premature Fc-GLP1 fusion protein secretion from the medial Golgi [29] and/or interference with normal Fc glycan processing by the engineered PAM proteins.

In order to facilitate analysis of α-amidation, the purified fusion proteins were cleaved using the engineered site inserted into the linker region connecting human IgG1 to GLP1-Gly and the mass of the released peptide was determined by mass spectroscopy (Table 2). The expression of exogenous PAM increased the extent of peptide amidation from 71 % in cells only expressing endogenous CHO PAM to 90 % or more in all of the lines synthesizing exogenous PAM (all *p* ≤ 0.011). PAM-TGN and PAM-ER cells produced amidated GLP1 significantly more effectively than did PAM1 cells (*p* < 0.05), which suggests that slowing the progression of membrane PAM through the cell improves the efficiency of the amidation process, achieving one goal of these studies.

**Table 2:** Mass spectroscopic determination of amidation of GLP1 by various PAM proteins. Fc-GLP1 secreted by the indicated cell lines was purified using Protein A; after cleavage with the Rhinovirus protease [19], products were subjected to mass spectroscopic analysis; data for Fc-AP-GLP1 secreted by CHO cells expressing PAM1, PAM/ER, PAM/TGN and PAM/Endo are shown. Mean ± SEM

PAM source	% Amidation
Endogenous	71.4 ± 0.3
PAM1	89.5 ± 1.5
PAM/ER	93.2 ± 1.3
PAM/TGN	95.4 ± 2.2
PAM/Endo	92.6 ± 3.1

**Fig. 5:**
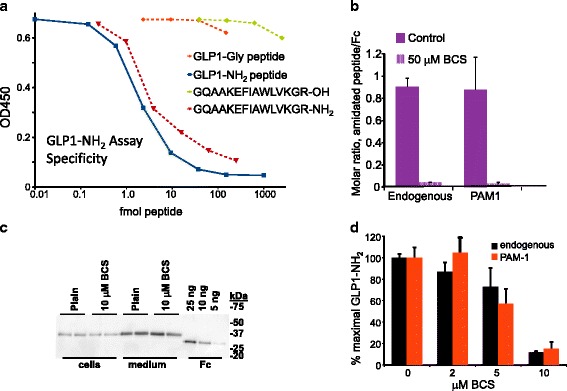
GLP1-amide enzyme immunoassay. **a**. Validation of GLP1-amide assay using synthetic peptides. The amidated peptide representing the C-terminal half of GLP1 diluted in parallel with the GLP1-NH_2_ standard, as did culture medium from all the CHO cell lines producing Fc-GLP1-Gly (not shown). Both the Gly-extended GLP1 and the des-Gly peptide (terminating in Arg) showed no cross-reactivity in the assay. **b**. CHO cells expressing AP-GLP1-Gly (no exogenous PAM) or AP-GLP1-Gly and PAM1 (PAM1) were plated onto 12-well plates and incubated with control medium or medium containing 50 μM BCS for 16 h; spent media and cells were harvested and subjected to Western blot analysis for Fc and EIA for GLP1-NH_2_. The molar ratio of GLP1-NH_2_ to Fc is shown. **c**. Western blot for Fc from one of the experiments included in (**c**), demonstrating the lack of toxicity of the BCS treatment. **d**. Dose response for BCS, demonstrating the similar effect of BCS on the ability of endogenous CHO PAM and exogenous PAM1 to produce amidated GLP1-NH_2_; average of 2 assays

To expedite analysis of Fc-GLP1-Gly amidation, we explored the use of enzyme-linked immunoassays (EIA). A solid phase EIA for GLP1 was shown to be specific for the amidated peptide (Fig. 5a); neither the GLP1-Gly precursor nor a peptide terminating with -Arg-OH instead of –Arg-NH_2_ was recognized. Use of the GLP1-NH_2_ EIA to assess Fc-GLP1 amidation in spent medium was validated by exposing CHO cells expressing Fc-GLP1-Gly without or with PAM1 to bathocuproinedisulfonate (BCS), a cell impermeant copper chelator specific for Cu(I) (Fig. 5b) [30]. After overnight (16 h) incubation of cells with 50 μM BCS, the molar ratio of GLP1-NH_2_ to Fc dropped more than 20-fold for both cell lines, demonstrating the utility of the EIA. Notably, secretion of the Fc-GLP1 fusion protein was not affected by the presence of BCS (Fig. 5c).

We next compared the ability of different concentrations of BCS to limit the amidation of Fc-GLP1 produced in CHO cells lacking exogenous PAM and in CHO cells expressing PAM1 (Fig. 5d). In both lines, Fc-GLP1 amidation was almost entirely prevented by incubation of the cells with medium containing 10 μM BCS. The sensitivity of endogenous CHO cell PAM and exogenous rat PAM1 to BCS was similar.

### Analysis of Fc-NMU and Fc-PYY fusion protein amidation

**Fig. 6:**
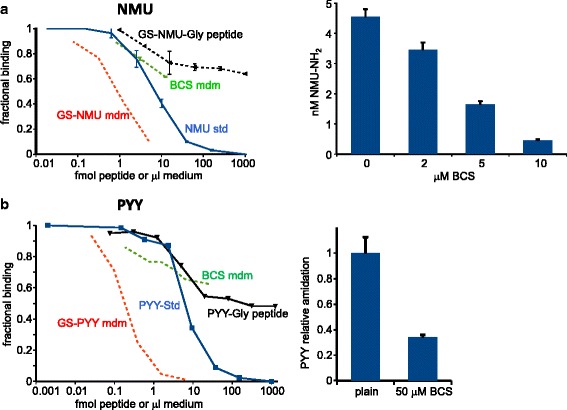
NMU and PYY enzyme immunoassays require amidation for full cross-reactivity. **a**. Characterization of NMU-amide assay using synthetic peptides. *Left*: the medium from PAM/820 s cells exhibited a dilution pattern parallel to the amidated standard peptide. Synthetic NMU-8-NH_2_ (Bachem) was almost identical to the full 25-residue NMU-NH_2_ in the assay (not shown). By contrast, the Gly-extended synthetic peptide and CHO medium from PAM/820 s cells after BCS treatment showed similar flat, partial dilution curves. *Right*: BCS in a dose-dependent manner inhibited the amidation of NMU by the PAM/820 s cells, using data from 1 to 6 μl of medium. Secretion of Fc-NMU was not affected (not shown). **b**. *Left*: Characterization of PYY-amide assay using synthetic peptides. Left: medium from CHO cells expressing PAM1 diluted in parallel with the authentic PYY-NH_2_ standard, while the Gly-extended peptide and medium from BCS-treated cells exhibited a much flatter dilution profile. *Right*: 50 μM BCS inhibited amidation of PYY, using data from 0.5 to 2 μl medium. For both NMU and PYY, data from several assays were normalized using their respective standards to enable the comparisons

Since Fc-NMU-Gly and Fc-PYY-Gly are amidated less extensively than Fc-GLP1-Gly when expressed in CHO cells [19], we evaluated the ability of commercially available EIA kits for these peptides to distinguish the amidated peptide from its precursor (Fig. 6). The NMU EIA recognized NMU8 and NMU25 equally, even though only the 8 C-terminal amino acids of NMU25 are present in NMU8. Longer NMU peptides with a –Gly residue appended to the –Asn that is normally amidated gave only partial cross-reactivity (Fig. 6a). Consistent with the fact that mass spectroscopic analysis revealed that ~70 % of the Fc-NMU-Gly secreted by CHO cells expressing only endogenous PAM was amidated [19], samples of spent medium from CHO cells expressing Fc-NMU-Gly with the Ala-Pro or the Gly-Gly-Ser linker generated competition curves parallel to those of amidated NMU. As for Fc-GLP1 amidation, growth of CHO cells in medium containing increasing concentrations of BCS eliminated the ability of the spent medium to generate competition curves parallel to that of the NMU standard; instead, partial competition was observed, with a curve resembling that generated by the GS-NMU-Gly peptide (Fig. 6a, *right*). Based on these observations, we concluded that amidated Fc-NMU fusion protein was no longer being secreted.

We next assessed the ability of an EIA for PYY to distinguish between amidated PYY and PYY-Gly (Fig. 6b). As for NMU, the assay for PYY showed partial cross-reactivity with PYY-Gly. Samples of spent medium from CHO cells expressing Fc-PYY-Gly generated curves parallel to those of amidated PYY and easily distinguishable from those of non-amidated PYY-Gly. Spent medium samples from CHO cells expressing Fc-PYY-Gly and kept in medium containing BCS generated competition curves more parallel to those generated by PYY-Gly than the full competition curve generated by amidated PYY (Fig. 6b); inclusion of 50 μM BCS in the medium largely abolished secretion of the fully reactive PYY peptide.

### Enhancing Fc-peptidylglycine amidation

**Fig. 7:**
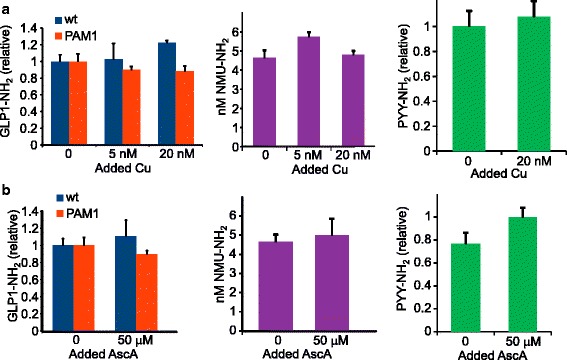
Varying copper and ascorbate concentrations. **a**. *Copper*. Adding small amounts of CuSO_4_ did not increase amidation of GLP1 by endogenous CHO cell PAM or by exogenous PAM1, the amidation of NMU by exogenous PAM/820 s, or the amidation of PYY by exogenous PAM1. The base medium contained 5 nM Cu (Life Technologies website). **b**. *Ascorbate*. Adding a concentration of ascorbate equivalent to normal plasma levels (AscA: 50 μM) [56] did not increase amidation of GLP1, NMU or PYY by the same cell lines as in (**a**). The base medium did not contain ascorbic acid. For all three peptides, data from several assays were pooled

Since PAM requires copper and consumes ascorbate, we explored the possibility that providing increased amounts of either required component might increase the ability of CHO cells to secrete amidated fusion protein. The concentration of copper in DMEM/F-12 is 5 nM (http://www.thermofisher.com/us/en/home/order/cell-culture-transfection-reagents.html). Although cultured cells maintained in concentrations of CuSO_4_ as high as 10 μM grow normally [30], addition of this level of exogenous CuSO_4_ to spent medium from CHO cells producing PHMcc demonstrated that PHMcc was rapidly inactivated (data not shown). We therefore asked whether a more modest increase in Cu levels in the culture medium promoted amidation (Fig. 7a). CHO cells expressing Fc-peptide-Gly were incubated in DMEM/F-12 medium containing 0, 5 or 20 nM additional CuSO_4_ for 16 h. Based on EIA data and Western blot analysis of the secreted product, the extent of amidation of Fc-GLP1, Fc-PYY and Fc-NMU was not altered by the addition of CuSO_4_.

Although CHO cells express gulonolactone oxidase (XM_003505165.1) and thus do not have the same genetic block to ascorbate synthesis present in humans and some other mammals, we next asked whether provision of additional ascorbate could increase the extent of Fc-peptide amidation. Cells were maintained in medium containing 0 or 50 μM ascorbate for 16 h; cells and media were harvested and EIAs and Western blots were carried out. The provision of additional ascorbate had no significant effect on the extent of amidation of Fc-GLP1-Gly, Fc-NMU-Gly or Fc-PYY-Gly (Fig. 7b). Blood levels of ascorbate are around 50 μM [31], and 50 μM ascorbate restores peptide amidation in rat pituitary cells maintained in serum-free medium [32].

## Discussion

Chinese hamster ovary cells are used to produce >70 % of all biopharmaceuticals [20]. It is now clear that CHO cells producing monoclonal antibodies catalyze their α-amidation to varying extents [20, 33–35]. Unlike monoclonal antibodies, where amidation could impair optimal functionality [20], we investigated methods that would maximize the amidation of Fc-peptidylglycine fusion proteins, wherein amidation would increase the biological potency of the product peptide. The only enzyme which can amidate the COOH-terminus of peptides is PAM [36, 37]. In most neuroendocrine cells the extent of peptide amidation is close to 100 %, even with only one functional allele of PAM [38, 39]. In neuroendocrine cells, most peptide amidation occurs in large dense core vesicles, which are stored for periods of many days, allowing plenty of time to complete the amidation process [40]. CHO cells lack such long-term storage sites, so PAM was targeted to various intracellular sites in an attempt to increase the access of PAM to its peptidylglycine substrates and maximize the extent of amidation.

Targeting PAM to different locations in the cell turned out to be more difficult than anticipated. When preceded by the PAM signal sequence, expression of the protease resistant catalytic cores of PHM and PAL yields active enzyme that is rapidly secreted by CHO cells [41] or efficiently stored in the secretory granules of AtT-20 cells [28]. In contrast, PAM1 is largely localized to the TGN area in both cell types; in the perinuclear region of AtT-20 cells, PAM1 has been identified in vesicular components of both the endocytic and biosynthetic pathways [42]. Truncation of PAM1 immediately after the stop transfer signal that follows its transmembrane domain (PAM1/899) yields a protein that cannot undergo endocytosis and accumulates on the cell surface [43]; this observation highlights the essential role played by endocytic trafficking in localizing membrane PAM. The fact that PAM1 fused to ER, endosomal or TGN targeting signals localized primarily to the perinuclear *trans-*Golgi network region is consistent with a major role for the luminal domains of PAM in controlling its endocytic trafficking [44]. A strong influence of the luminal domain on efforts to redirect proteins to novel subcellular locations is well established [25]. The specific activity of PHM did not differ when it was part of PAM/TGN, a TypeII integral membrane protein or part of PAM1, PAM/ER, PAM/Endo or PAM/Lyso, which are all Type I integral membrane proteins (Fig. 1c).

Each method for determining the extent of peptide amidation has its advantages and drawbacks. The mass spectrometry approach provides data for both the nonamidated and amidated peptide in a single run, but the relative recovery of the two peptides (−Gly and –NH_2_) is difficult to assess without synthetic standards for every case. The EIA is very selective for the amidated end (especially the GLP1-NH_2_ EIA) and Western blots allow assessment of protein stability. The large dynamic range of the Genegnome facilitates analysis, but the dilutions needed for the EIA complicate the analysis.

Our data suggest that PAM speeds the rate of trafficking of the Fc-peptides through the secretory pathway, at least for the PAM1 and PAM/TGN lines (Table 1). This change may parallel the changes seen in the trafficking of soluble products derived from pro-opiomelanocortin in pituitary corticotropes, where expressing PAM1 increases the rate of basal secretion [45]. Like chromogranin A, PHM and PAL are efficiently targeted to secretory granules in corticotrope tumor cells. While expression of exogenous chromogranin A in non-endocrine cells causes the formation of granule-like structures [46], neither expression of PHMcc, PAM820s or PAM1 caused granule formation in CHO cells.

Although extensive amidation of the Fc-AP-GLP1-Gly substrate occurred in cells expressing only endogenous CHO PAM, expression of exogenous PAM1 or targeted PAM did significantly increase the percentage of substrate that was amidated. Efforts were made to determine what factors control the extent of amidation of the Fc-peptide substrates. As expected, removal of copper blocked amidation. Although copper-dependent, PAM does not bind copper tightly, making it susceptible to copper chelators. This raised the possibility that increasing copper delivery to PAM could increase peptide amidation. However, the nanomolar levels of copper in DMEM/F12 (5 nM) appear to be adequate; raising the copper concentration in the medium five-fold had no effect on the extent of amidation. Higher levels of copper inactivated PHM and oxidized the secreted Fc peptides.

Since CHO cells express gulonolactone oxidase, they can synthesize ascorbate and are not dependent on acquiring it from the medium; consistent with this fact, supplying additional (exogenous) ascorbate did not increase the extent to which any of the Fc-peptidylglycine substrates was amidated. In contrast, all human cells and rodent neurons and endocrine cells rely on ascorbate taken up from their environment. DMEM/F12 does not contain ascorbate, and peptide amidation by rat pituitary cells maintained in serum-free medium is dependent on exogenous ascorbate [32].

## Conclusions

Elimination of CHO cell PAM expression and activity would avoid unintended amidation (e.g. of antibodies), which may have a therapeutic benefit (if amidated antibodies are mildly antigenic, for example) [20]. However, there are apparently no adverse consequences of having a portion of the monoclonal antibodies amidated, since that is also true of antibodies produced endogenously [33, 35]. The point of these studies was to determine whether CHO cells expressing PAM and a Fc-peptide substrate could be made to amidate a high percentage of the Fc-fusion peptide, for the purpose of producing biopharmaceuticals, and the answer is quite clearly in the affirmative. The broad specificity of PAM and its ability to function when traversing the cell surface or when targeted to the endocytic pathway raise the possibility of using PAM to catalyze other modifications of exogenously supplied substrates.

## Methods

### Molecular biology

The pCIS vector encoding rPAM1 was described [47]. To construct a pCI.neo vector encoding PAM/GFP, residues D^408^VH—RDR^462^ of rPAM1 were replaced by a linker (EDPRPVAT) followed by enhanced green fluorescent protein (EGFP), which has the non-dimerizing A^206^K mutation [22]. A stop codon was introduced into rPAM1 and PAM/GFP after -RSV^820^ to produce the soluble proteins PAM820s and PAM820s/GFP, respectively. Residue -SGR^911^ of PAM/GFP was fused to -GKKAA (modeled after the ER retention signal in ERGIC-53) [23] to yield PAM/ER. PAM/TGN was created by inserting residues M^1^RL—PGP^110^ of the TGN-localized enzyme hGalactosyl Transferase 1 (AAH45773.1) immediately before residues S^42^FS- of PAM820s/GFP [24]. PAM/Endo was made by fusing PAM820s/GFP through an *Xba1* linker encoding –SR- to residues S^184^HS—LPM^277^ of hCation Independent Mannose-6-Phosphate Receptor (NP_002346.1), which traffics through the endosomal compartment [25]. PAM/Lyso was created in a similar manner, with the *Xba1* linker connected to D^378^EN—QTI^417^ of hLAMP-1 (NP_005552.3), an enzyme targeted to lysosomes [48]. All constructs were verified by DNA sequencing. The Fc-linker peptide substrates with blasticidin drug selection were described [19], and contain linkers (GS: (GGS)_6_-GGLEVLFQGP and AP: (AP)_10_-LEVLFQGP) between the Fc and the peptide substrates.

### Cell culture

pEAK Rapid cells (hEK-293 derivative; Edge Biosystems, Gaithersburg MD) were transiently transfected using Lipofectamine 2000 (Invitrogen Life Technologies, Grand Island, NY) as described [49]. To test the efficacy of the targeting signals, vectors encoding targeted PAM were expressed transiently in AtT-20 corticotrope tumor cells plated onto coverslips coated with poly-L-lysine followed by NuSerum (Corning, Corning NY). Cells on coverslips in 24-well dishes were transfected with 1 μg DNA complexed with 2.5 μl Lipofectamine 2000. Cells were fixed with 4 % formaldehyde in phosphate buffered saline approximately 24 h after transfection.

Stable clones of CHO cells expressing PAM1 and PAM820s from the pCIS vector were selected in Alpha-MEM with dialyzed fetal bovine serum [2]. Stable clones of CHO cells expressing PAM/GFP, PAM/ER, PAM/TGN and PAM/Endo encoded by a pCI.neo vector were established in DMEM/F-12 containing 10 % fetal bovine serum and 0.5 mg/ml G-418 [50]. Stable clonal PAM/Lyso lines were never successfully selected. Other cell lines expressing Fc-peptide precursor fusions were established previously [19]. For each clonal line examined and reported in depth, there were 2–6 additional lines with lower expression levels that exhibited similar properties.

Medium levels of Cu and ascorbate were adjusted using 100-500X stocks of ascorbate, CuSO_4_, and bathocuproinedisulfonic acid (BCS); stocks were sterile filtered and added to the medium 24 h before the start of a 16 h collection. Secretion of Fc-fusion peptides was detected using a peptide-specific Enzyme ImmunoAssay (EIA) and by Western blot analysis for Fc.

### Biochemistry

PHM and PAL activity were assayed in duplicate using [^125^I]-labeled substrate (Acetyl-Tyr-Val-Gly), 0.5 μM unlabeled substrate and multiple sample dilutions as described [51]. Western blots were performed as described using antisera to PHM (JH1761), GFP, γ-adaptin (#61038; BD Transduction Laboratories) or human Fc and HRP-tagged secondary antisera [19].

Enzyme ImmunoAssay kits specific for the indicated peptides were purchased from Bachem (Torrance, CA): GLP1(7–36) amide [S-1141]; GLP1 (7–37)Gly [S-1216]; Neuromedin U-25 [S-1253]; PYY [S-1151]. Spent medium samples were diluted 1:5 in EIA buffer before being assayed. Samples were further diluted into buffer based on Western blot data for Fc concentration. Dilutions of the standard peptide for each assay were prepared per the manufacturer’s instructions. One well did not receive any standard peptide and defined maximum binding. One well did not receive antibody and defined minimum binding. Standards and samples were added to strips of a 96-well plate coated with antibody to rabbit IgG in a final volume of 50 μl. Dilute antibody (25 μl) was added to each well except the no antibody control and samples were incubated for 1 h at RT. Biotinylated peptide (25 μl) was added to each well and samples were incubated for 2 h at RT. Wells were washed five times with 200 μl buffer. Streptavidin-tagged HRP (100 μl of a 1:600 dilution in buffer) was added to each well and the samples were incubated for 1 h at RT. After washing again as described, substrate (100 μl of TMB diluted three-fold with 100 mM Na acetate, pH 5.0) was added. After ~4 min, the reaction was stopped by adding 110 μl HCl (2 M) to each well and absorbance was read at 450 nm using a Wallac plate reader (Victor 1420). Peptide concentrations in unknown samples were calculated using the weighted logit-log method [52]. Analysis of synthetic GLP1-NH_2_ and GLP1-Gly revealed that the GLP1 (7–37)Gly [S-1216] assay cross-reacted fully with GLP1-NH_2_, so this assay was not used further.

### Immunostaining

Four-well chamber slides [Thermo Scientific, Waltham, MA] were coated with poly-L-lysine (0.1 mg/ml) to enhance cell attachment. Cells were fixed with 4 % formaldehyde [J.T.Baker, Center Valley, PA] in PBS (20 min) or with ice cold MeOH (20 min) depending on the antibody used.

Antisera to PAM were generated in-house and were described previously: mouse monoclonal 6E6 to the cytosolic domain (1:10 dilution of spent medium) [53]; JH1761, rabbit antibody to purified recombinant PHM (1:1000) [54]; JH629, rabbit antibody to exon 16 (1:1000) [55]; CT267, rabbit antibody to the C-terminus of PAM (1:1000) [50].

The following subcellular marker antibodies were used: calnexin (1:500; #208882; Millipore, Billerica, MA); GFP (1:1000; #75–131; NeuroMab, Davis, CA); giantin (1:1000; ab24586; Abcam, Cambridge, MA); early endosomal antigen 1 (EEA1) (1:500; #324610; Calbiochem, Billerica, MA); human Fc (1:4000; #309–001–008; Jackson ImmunoResearch, West Grove, PA); human Fc (1:100; #9042–01; SouthernBiotech, Birmingham, AL). Secondary antibodies (all from Jackson ImmunoResearch, West Grove, PA) were: FITC-tagged donkey anti-mouse (1:500; #715–096–151); FITC-tagged donkey anti-rabbit (1:500; #715–096–151); Cy3-tagged donkey anti-rabbit (1:2000; #711–166–152).

### Animal ethics

The work used no animals, only established cell lines.

## Abbreviations

CHO: Chinese hamster ovary
GLP1: Glucagon-like peptide 1
NMU: Neuromedin U
PAL: Peptidyl-α-hydroxyglycine α-amidating lyase
PAM: Peptidylglycine α-amidating monooxygenase
PYY: Peptide YY
